# *Plantago media* L.—Explored and Potential Applications of an Underutilized Plant

**DOI:** 10.3390/plants10020265

**Published:** 2021-01-30

**Authors:** Radu Claudiu Fierascu, Irina Fierascu, Alina Ortan, Alina Paunescu

**Affiliations:** 1 National Institute for Research & Development in Chemistry and Petrochemistry—ICECHIM, 060021 Bucharest, Romania; fierascu.radu@icechim.ro; 2 Department of Science and Engineering of Oxide Materials and Nanomaterials, University “Politehnica” of Bucharest, 011061 Bucharest, Romania; 3 University of Agronomic Sciences and Veterinary Medicine of Bucharest, 011464 Bucharest, Romania; alina_ortan@hotmail.com; 4 Department of Natural Sciences, University of Pitesti, 1 Targu din Vale Str., Pitesti, 110040 Arges, Romania; alina_paunescu@yahoo.com

**Keywords:** *Plantago media* L., composition, biomedical applications, potential uses

## Abstract

The search of valuable natural compounds should be directed towards alternative vegetal resources, and to the re-discovery of underutilized plants. Belonging to the *Plantaginaceae* family, the hoary plantain (*Plantago media* L.) represents one of the lesser studied species from the *Plantago* genus. The literature study revealed the under-utilization of the hoary plantain, a surprising aspect, considering its widespread. If the composition of *Plantago media* L. is rather well established, its applications are not nearly studied as for other *Plantago* species. The goal of the present paper is to summarize the findings regarding the applications of *P. media,* and, having as starting point the applications of related species, to propose new emerging areas of research, such as the biomedical applications validation through in vivo assays, and the evaluation of its potential towards industrial applications (i.e., development of food or personal care products), pisciculture or zootechny, phytoremediation and other environmental protection applications, or in the nanotechnology area (materials phytosynthesis). The present work constitutes not only a brief presentation of this plant’s present and potential applications, but also an invitation to research groups world-wide to explore the available vegetal resources.

## 1. Introduction

Accompanying the development of human civilization, plants were commonly used as food, feed or for empirical medicinal purposes [1]. The development of the modern medicine led to the loss of important ethnomedicinal data, accompanied by the disappearance or the reduction of the growing area of medicinally important plants [2]. However, the last decades led to the resurrection of alternative, plant-based medicine [3], together with a search of alternative, “bio” products [4], as well as the discovery of new potential applications of the vegetal materials [5]. Several plant-originating biomolecules have even pursued the long road from “plant to pharmacy shelf”, resulting in economically important commercial products [6]. With the identification of commercially valuable phytochemicals, the vegetal resources could become the subject of over-harvesting, producing environmental or ecological imbalances [6]. This could be avoided by continuously searching for alternative vegetal resources, and by the re-discovery of underutilized plants.

The *Plantaginaceae* family contains herbs or small shrubs, their habitat ranging from terrestrial to aquatic. The family contains only one genus and approximatively 270 species [7]. *Plantago* genus is characterized by a wide variety of component phytochemicals, but the most encountered are the iridoid glucosides, flavonoids, hydroxycinnamic acids, terpenoids, polysaccharides, unsaturated fatty acids, vitamins, alkaloids, terpenes and saponins (leaves), respectively xylose and galacturonic acid (mucilaginous seeds) [8,9]. The genus is world-wide represented, several species having a weedy character [7].

Belonging to the *Plantaginaceae* family, the hoary plantain (*Plantago media* L.) represents one of the lesser studied species from the *Plantago* genus. Native to Eurasia and introduced in most parts of the world [10], the plant is a perennial herb, characteristically growing on chalk or limestone soils, but often also encountered on heavy clay soils. Its habitat is mainly related to the presence of a calcium source, being encountered mainly on downland grassland, calcareous pasture or even in water-meadows beneficiary of calcareous water [11]. The species is morphologically characterized by the slender stalk (5 to 50 cm), basal, finely-haired elliptic to ovate leaves, developing in rosette pattern, over 3 cm wide, presenting 7–9 veins and equipped with midribs that can be easily separated from the mesophyll tissue, curly, abundant, or sparsely scattered lamina trichomes on both epidermis, a petiole shorter than the leaf lamina, delicate pink-white flowers (appearing May–September) that are pollinated by wind and insects, and contains 4 seeds per capsule [12,13,14,15,16,17,18,19]. A tetraploid species, *P. media* shows treading resistance, a feature related to the resistance being represented by the strong root contraction [10]. The hoary plantain is edible (fresh young leaves being used in fresh salads or cooked as other leafy green vegetables) [20] and its uses were apparently common in the past, its seeds being encountered in the archaeological excavations from Roman period Britain [21,22] and even earlier [23]. Its use in folk medicine included several applications, such as antimicrobial, anti-inflammatory, anti-histaminic, hemostatic, cicatrizing, expectorant and diuretic [20,24]. The commonly used part for medical purposes is the leaves, used for the preparation of infusions [22].

The present work aims to summarize the scientific findings regarding *P. media* composition and potential uses, based on published research data, highlighting the importance of this natural resource. The methodology for the article collection contained the survey of the main scientific data-bases (Scopus, Web of Science, ScienceDirect, and PubMed), using as specific keyword “*Plantago media*”. The validation of the articles was performed manually (by reading the entire article). Another important aspect covered by the present work is represented by the proposal of new emerging areas of research for *P. media* (by comparison with related species), such as the development of food or personal care products, the use in pisciculture or zootechny, phytoremediation and other environmental protection applications, or in the nanotechnology area (materials phytosynthesis). 

## 2. Main Constituents and Applications

### 2.1. General Composition

As previously mentioned, the composition of other *Plantago* species (*P. lanceolata, P. major, P. ovata*) is well-established and known, subject of several review papers [25,26,27,28]. *P. media,* in turn, did not receive such attention from the scientific community, no review paper presenting the species composition being identified. As a genus characteristic, the most important components of the hoary plantain are the polysaccharides [29]. Ollenikov et al. identified in the *P. media* extracts several important polysaccharides (galactose, arabinose, xylose, mannose, glucose, as well as trace amounts of rhamnose and fucose) [30] (Table 1). It is worth to be noted that, according to some authors, the total polysaccharide content was found to be the highest in *P. media,* compared with other species [31]. 

Some studies also evaluated the composition of *P. media* in terms of total sterol esters, total lipids and total fatty acids, also identifying several individual components [32]. Total fiber and lipid content were also evaluated by other groups [33], revealing a relatively low amount of lipids and a total fiber higher than the level recorded for vegetables like beets or spinach (2.25 g/100 g fresh weight). The study also quantified the presence of other compounds (vitamin C, oxalic acid) and minerals (Na, K, Ca, Mg, P, Fe, Cu, Zn, Mn), *P. media* revealing higher levels in K, Ca, P, Fe, Cu, and Zn, compared with other *Plantago* species (*P. major* and *P. lanceolata*) [33], suggesting that *P. media* could represent a valuable source of minerals. Total available carbohydrates were also evaluated, suggesting a low content for *P. media* (1.99 g/100 g fresh leaves) [33].

Although several alkaloids were identified in other *Plantago* species (indicain and plantagonin in *P. major* [34], dictyoquinazol C and sampangine in *P. ovata* [35]), no alkaloids were identified in the published studies regarding *P. media*.

The general composition of *P. media* can be completed with the presence of other documented compounds, such as iridoids (aucubin, melittoside, monomelittoside, 10-acetylmonomelittoside, 10-acetylaucubin, catalpol), hydroxycinnamic acids (caffeic, chlorogenic, ferulic, gallic, neochlorogenic acid isochlorogenic acids), flavonoids (luteolin, apigenin, kaempferol), glycosides (verbascoside, plantamajoside, homoplantaginin), phytosterols (campesterol, stigmasterol, sitosterol), saturated and unsaturated fatty acids (linoleic acid, hexadecatrienoic acid, palmitic acid, myristic acid, palmitoleic acid, behenic acid, erucic acid) and carotenoids (β-carotene, violaxanthin, lutein, neoxanthin, zeaxanthin), the latter having an important photoprotection role for the photosynthetic apparatus, leading to an increase resistance to excess solar radiation [36]. Some authors [8] designate the aucubin-related iridoids levels (aucubin, catalpol, 10-O-acetylaucubin, monomelittoside, 10-acetylmonomelittoside, melittoside) as species-specific, differentiating the hoary plantain among others *Plantago* species.

The above-presented compounds only show a general composition of the species, the actual levels and, in some cases, the presence/absence of some of the minor compounds varying in the literature data. As for any plant material, the phytoconstituents levels in the plant extracts are dependent on several factors (including, but not limited to ecological factors, zoning, culture technology, or processing methods) [6]. For example, as a response to environmental conditions, several morpho-physiological parameters (such as specific leaf area density and organ mass) were found to be modified in a comparative study on two hoary plantain populations by Rozentsvet et al. [44]. The changes were also reflected in different lipids and fatty acids content, that also varied with the ontogenetic stage. 

*P. media* was defined as “salt-sensitive”, its exposure to up to 200 mM NaCl leading to a reduction of shoot length and stomatal conductance, as well as in a decrease of superoxide dismutase (SOD), catalase (CAT), glutathione reductase (GR) activities with increasing salinity, suggesting a sensitive structural arrangement in leaves of the hoary plantain against stress [45]. On the other hand, the plant is resistant to excess solar radiation, a character associated with the presence of the carotenoids, their resistance being attributed to the activation of non-photochemical quenching mechanisms associated with the xanthophyll cycle de-epoxidation [36]. As such, the levels of photosynthetic pigments are higher in the plants grown in the shade, compared with those grown in high light conditions (up to two times higher) [18,40]. 

The different types of other compounds identified in the composition of *P. media* also have important roles in its development. Phenolic compounds represent the most important class of natural antioxidants, multiple studies presenting the direct correlation between the phenolic content and the antioxidant activity of different plant tissues [46]. 

The iridoid glycosides present in the leaves of *Plantago* (their level being correlated with the plant’s ontogenetic stage) are also part of the plants’ defense mechanisms against external factors (pathogens or non-adapted herbivores), as demonstrated for other *Plantago* species [47,48]. The phytosterols are related to the humidity adaptation of the plants, pathogens defense or regulation of the membrane fluidity and permeability [49,50]. 

The polysaccharides present in the seed coats of *Plantago* species swell in contact with water, forming a high-viscosity mucilage; the mucilage facilitates the attachment of seeds to humans and animals, and thus contributing to the spread of the plant [51]; it also protects myxospermatic diaspores in the passage through the digestive system of birds [52]. 

Flavonoids and phenolic acids, accumulating in the plant tissues in response to various biotic and abiotic stress, ensure the adaptation of the plant, through multiple physiological functions, while also having other roles, such as growth regulation, pigmentation, respectively precursor molecules for other physiological important compounds [53].

Fatty acids are commonly used throughout the plant kingdom as a source of carbon and energy [54], while vitamin C, besides its well-known antioxidant effect, also has multiple roles in the plant physiology, as recently presented by Paciolla et al. [55].

All the above-mentioned compounds were found to possess important biological properties, which is expected to reflect in the future studies regarding the properties of *P. media* extracts.

### 2.2. Documented Applications

Generally speaking, plants contain several types of compounds which provides good antioxidant activity [56], such as phenolic acids, flavonoids or terpenes. In recent years, the focus of both scientific community and of the desire for healthier foods led to the exploitation of natural resources for the separation of natural antioxidants as viable alternatives for the synthetic additives [56]. The antioxidant character of individual compounds or mixtures (such as natural extracts) allows their application in food industry (to delay the autooxidation, neutralize free radicals) [57] or in cosmetic industry (i.e., by harvesting their protecting effect for the skin from photoaging) [58]. Indirectly, as the oxidative stress represents a major factor related to the apparition of several diseases (cardiovascular, neurodegenerative, oncological, etc.), the antioxidants could contribute to the development of scientifically solid nutraceuticals [57].

The antioxidant capacity of natural extracts can be evaluated by a series of in vitro or in vivo assays, each one having their advantages and shortcomings. As a general remark, the in vitro assays should be considered as a “preliminary test”, with little biological relevancy, whose conclusions should be verified by in vivo assays [56]. However, as those preliminary assays are easily accessible, inexpensive and require minimal instrumentation, most pioneering works for vegetal species are performed in vitro. This is also the case of *P. media,* whose antioxidant activity was firstly reported by Beara et al. [59] in a comparative study, involving several antioxidant assays, registering an average, assay-dependent antioxidant capacity, by comparison with other *Plantago* species and with the standard 3,5-di-tert-butyl-4-hydroxytoluene (BHT). For example, the extract had a good antioxidant activity in the DPPH assay (2,2′-diphenyl-1-picrylhydrazyl radical reduction assay) and, especially, FRAP (ferric reducing antioxidant power) assay, while poorly performing in the others assays [59]. The correlations identified by the authors between the total phenolics, respectively, total flavonoid contents and the results of the antioxidant assays were satisfactory only for the DPPH and FRAP assays (R^2^ > 0.94), suggesting the involvement of other compounds in the scavenging of the radical species used for the assays [59]. The quantification of the two types of phytoconstituents revealed that the evaluated *P. media* extract had the highest phenolics content (compared with extracts obtained by the same procedure from *P. argentea,* P. *holosteum*, *P. major*, and *P. maritima*), and an average flavonoids content (higher than *P. argentea* and *P. major,* lower than *P. holosteum* and *P. maritima*). Similar results were obtained by Gonda et al. [60] in the CUPRAC (cupric reducing antioxidant capacity) assay (see Table 2), classifying *P. media* as an average antioxidant source, under the values obtained for *P. lanceolata* and *P. maritima,* although superior to the more studied *P. major*, or *P. altissima.* The authors identified a direct correlation between the total phenylethanoid content (higher than *P. altissima, P. major*, and equal to *P. lanceolata*) and the antioxidant potential. The aucubin content was found to be lowest among the studied extracts, equal to the one in *P. major* [60].

The results obtained by Grigore et al. [61] in the antioxidant enzyme activity assays suggest a higher antioxidant potential of *P. media* (compared with other *Plantago* species), and a superior activity in the flowering phenophase. Lukova et al. also obtained superior results in the in vitro antioxidant assays for the hoary plantain, compared with *P. major* and *P. lanceolata*, although significantly lower compared with the values obtained for the BTH standard, for ethanol extracts, hemicellulose, respectively xylanase hydrolysates) [31,62].

The hydroalcoholic extract of *P. media* also proved to have high antioxidant capacity, even when compared with known medicinal plants (such as woundwort—*Anthyllis vulneraria* L. or wild thyme—*Thymus serpyllum* L.) [63]. The hydroalcoholic extract also exhibited superior antioxidant properties (DPPH and 2,2′-azino-bis(3-ethylbenzothiazoline-6-sulfonic acid—ABTS assays) compared with *P. cornuti*, *P. lanceolata* and *P. major*, at the same time showing no pro-oxidant properties (in the hemoglobin-oxidation in the presence of laccase) The total phenolics content of the extract was also found to be correlated with the results of the antioxidant assays (the highest among the four *Plantago* species studied), underlying the importance of this class of phytoconstituents for developing antioxidant recipes [64]. 

The first identified report regarding the potential biomedical applications of hoary plantain is represented by the study of Kunvári et al. [41] regarding the antitumoral potential of two glycosides isolated from the plant. The study revealed an important antitumoral potential of the compounds, exhibited through the significant inhibition of isolated EGF-R tyrosine kinases. 

The polysaccharidic fraction of the *P. media* was proven as an efficient anti-atherogenic agent, recording a 42.77% alkaline phosphatase binding (compared with the heparin control) [30], while the alcohol and lyophilic extracts presented mycostatic activity to several yeast and fungi strains (superior for the alcohol extract) best results being obtained against *Aspergillus oryzae* (clinical strain). The effect can be correlated with the levels of quantified polyphenolics (flavonoids, hydroxycinnamic acids) in the lyophilic extract, the highest content being recorded for verbascoside (2.013%), plantamajoside (1.723%), respectively ferulic acid (1.526%). [37].

Hydroalcoholic (80% methanol) extract of *P. media* showed inhibition activity of prostaglandin E2 (PGE_2_) and thromboxane (TXA_2_) eicosanoids production (similar to aspirin) at low-dose concentration (especially for PGE_2_), supporting future investigation of the species as a potential anti-inflammatory agent. In the same time, the extract did not present any significant cytotoxic potential. The studied extract revealed high levels of caffeic acid phenolic acids, flavonoids and triterpenic acids; also, the *P. media* showed the highest level of aucubin (44.272 mg/g) from the studies species (*P. altissima*, *P. argentea*, *P. holosteum*, *P. lanceolata*, *P. major*) [43].

Table 2 summarizes the biomedical properties of *P. media,* as presented in the cited literature data. 

## 3. *Plantago media* L. Future Perspectives 

The potential applications of *P. media* will be detailed in regard to other potential applications of *Plantago* species, in respect to recent literature data published. 

### 3.1. Health Applications

Supplemental applications of the *P. media* products in the health area could be suggested by other *Plantago* species properties. Several review papers present the potential uses of, in example, *P. lanceolata*, *P. ovata* or polysaccharide from *Plantago* sp. in this area [27,29,65,66]. These works could constitute a good starting point for the development of new nutraceuticals based on *P. media.* As previously presented (as the main application already studied, although trough in vitro assays—Table 2), the hoary plantain represents a potential source of antioxidant compounds; this is also confirmed by the study of other *Plantago* species, several of them possessing antioxidant potential (Table 3). Either if there are discussed aqueous, alcoholic, or organic extracts, polysaccharide fraction or mucilage, *Plantago* sp. (*P. albicans, P. coronopus, P. lanceolata, P. major, P. ovata, P. squarrosa*) exhibited good antioxidant potential in in vitro assays.as 

*P. squarrosa* and *P. major* L. exhibited anti-microbial potential [67,68] against several gram-positive and gram-negative bacteria or fungi, a good support of the mycostatic potential observed for *P. media* [37]. Another very important potential activity is represented by the antiviral potential. Chathuranga et al. [69] evaluated the antiviral potential of *P. asiatica* and its component verbascoside (acteoside), a compound that, as previously presented, can be found in relatively high quantities in *P. media* [37], against the respiratory syncytial virus, the in vivo assays suggesting a possible anti-viral path to be followed.

The anti-inflammatory potential of *P. media* [43] was supported by the application of *P. major and P. lanceolata* extracts as anti-inflammatory agents (either in vitro, in carrageenan-induced paw edema model, by determining the expression of the proinflammatory enzyme, cyclooxygenase, or on oral epithelial cells), an effect attributed to the presence of phenylethanoid compounds (in particular, verbascoside) [70,71,72,73].

An anti-tumoral potential was observed for *P. major* and *P. lanceolata* extracts [74,75,76], as well as for the polysaccharide fraction of *P. ovata* [77]. All the previously presented activities for different *Plantago* sp. represent a good indicator, supporting the reported applications of *P. media*. 

Other studies, in turn, would suggest potential applications of the hoary plantain that are waiting to be explored. For example, a hepatoprotective action was observed for the defatted aqueous methanolic extract obtained from the leaves of *P. major* (effect attributed to verbascoside) [70], *P. ovata* husk mucilage [78] and seed aqueous extract [79], *P. asiatica* seeds polysaccharide fraction [80], *P. psyllium* seeds ethanolic extract (for which the total phenolics and total flavonoids contents were determined as 16.17 mg gallic acid equivalents/g dried weight, respectively 1.9 mg rutin equivalents/g dried weight) [81] and *P. albicans* leaves aqueous extract (total phenolics content 592.75 mg gallic acid equivalents/g, total flavonoids content 116.7 mg catechin equivalents/g [82], in several hepatic damage models, Table 3). Considering the variety of extracts and fractions used, the results would suggest a hepatoprotective potential for the *P. media,* also.

The renoprotective effect of the *P. major* Soxhlet extracts (ethanol, 70%) was evaluated in Cisplatin and Adriamycin induced renal dysfunction in animal models [83,84,85], while the *P. albicans* leaves extract and *P. asiatica* and *P. depressa* (a species to which, according to recent phylogenetic analyses, *P. media* is closely related [19]) seeds extracts proved to have an anti-obesity potential, by effectively improving lipid and glucose metabolism in high-fat diet-induced obese mice [86,87,88].

The flavonoid fraction isolated and the leaves extract obtained from *P. major* (a species that, as previously stated [59], presents a lower total flavonoids content, compared with *P. media*), were evaluated as antiarrhythmic agents (by functional modulation of Na^+^ and Ca^2+^-channels in cardiomyocytes) [89], respectively as anxiolytic agents [90]. Arabinoxylan (a polysaccharide isolated from different *Plantago* sp.) proved to have anti-diabetic (by improvement of carbohydrate, lipid and amino acid metabolism) [91] and prebiotic (by enhancing the growth and antimicrobial activity of *Lactobacillus casei*) properties [92].

The *Plantago asiatica* L. extract and polysaccharide fraction were proved to have antihypertensive effect (trough angiotensin-converting-enzyme 46 inhibition, while simultaneously protecting organ damage against hypertension) [93], respectively to alleviates nonylphenol induced reproductive system injury (via PI3K/Akt/mTOR pathway) [94].

The whole plant extract of *Plantago rugelii* Decne was evaluated by Ogbiko et al. [95] in an anti-ulcer study, the results suggesting that the infusion (200 and 400 mg/kg) had a protective effect against gastric ulceration (induced by aspirin and HCl). Similar results were obtained by Bagheri et al. [79], using the aqueous *P. ovata* seeds extract, in an indomethacin-induced rat model, observing a reduction in microscopic and macroscopic ulcer index. Seed mucilage of *P. ovata* was used by Basiri et al. [96] as a potent lead biosorbent (increasing fecal excretion and decreasing lead tissue absorption) in mice models.

All these potential applications of *Plantago* sp. remains to be studied for *P. media,* as no studies in those area were performed to this date, up to our knowledge. 

### 3.2. Other Applications

Besides the health-related applications, *Plantago* sp. were evaluated for a series of industrial applications. The methanol extract of *P. lanceolata* could find application in fish farming, as its application was proven to promote growth, as well as to enhance immune responses and antioxidant enzyme activities in rainbow trout [97], while verbascoside and aucubin was proved to reduce NH_3_ production on rumen fermentation, reducing the N losses in the urine [98].

*P. lanceolata* extracts were proposed for the development of natural cosmetics (due to their UV protecting activity, as well as skin regeneration stimulation) [99], while the gum isolated from *P. major* seeds proved to have emulsifying and foaming properties, which supports the use of the fraction as an alternative hydrocolloid for emulsion and foam-based foods [100]. Related to the food industry, *P. major* mucilage (extracted either by hot-water extraction or ultrasound assisted extraction) was used for the development edible and biodegradable films [101,102]. This could lead to the development of bioproducts for increasing the shelf-life of meat products. For example, the application of the edible film (with a 1.5% dill essential oil content) increased the shelf life of beef by 9 days [101]. 

The mucilage separated from *Plantago* sp. could also find application in other important areas, such as scaffolds for cell culture, drug delivery systems or food additives. This would involve the development of biocompatible materials, such as those proposed by Allafchian et al. [103], based on *P. ovata* mucilage and polyvinyl alcohol. 

Correlated with their metal-uptake capacity, *Plantago* sp. could be used for phytoremediation potential. This application was studied, for example, for *P. lanceolata* and *P. major* for the removal of toxic heavy metals (Pb, As, Cd) [104,105]. The studies revealed a higher concentration of heavy metals in the roots, compared with the leaves, thus suggesting a limited mobility of the heavy metals, as a part of the resistance mechanism to heavy metals (involving an avoidance strategy, such as the immobilization of the metal at root level and in cell walls) [105]. The same plant was proved efficient in the phytoremediation of organic pollutants contaminated sites [106].

Another potential application of *P. media,* correlated with their metal up-take capacity could be in the improvement of mineral concentrations in the diet of livestock, to prevent the apparition of mineral deficiency, trough increasing species diversity in swards [107]. However, the hoary plantain affinity towards different metals could represent a drawback, as some studies [108] suggest a potential for *P. media* to up-take hazardous heavy metals. Although this aspect could be beneficial for phytoremediation strategies, it needs to be considered for other application, the control of heavy metals content in extracts should be performed before their application. The use of *P. lanceolata* in cattle diet was proven to reduce N_2_O emissions [109] and to increase the growth performance and carcass characteristics of lambs [110], areas in which *P. media* could find applications.

Another environmental application of *Plantago* sp. is represented by its mucilage ability to remove organic pollutants. The biocomposite membrane (*P. psyllium* mucilage, eggshell membrane and alginate) proposed by Mirzaei and Javanbakht [111] proved to have the ability to remove cationic and anionic dyes (methylene blue and methyl orange) from aqueous solutions, reaching an adsorption capacity of 5.45 and, respectively, 3.25 mg/g. Another potential application of the *Plantago* sp. is represented by their chemical inhibitor potential. For example, the polysaccharide fraction of *P. ovata* was proposed as a green corrosion inhibitor by Mobin and Rizvi [112], their study suggesting a protective effect of the developed material for the carbon steel in hydrochloric medium, presenting a good inhibition efficiency (92.53%) accompanied by a low risk of environmental pollution. The authors assign the main corrosion inhibitor role to the highly branched polysaccharide arabinosyl (galaturonic acid) rhamnosylxylan [112].

Finally, a new and promising application of *Plantago* sp. is related to the nanotechnology area, in particular for the nanoparticles phytosynthesis (synthesis of materials using plant extracts). Briefly, the phytosynthesis mechanisms involve the reduction of metals from metallic salts precursors to zero-valent nanoparticles or metallic oxides, using the different plant phytoconstituents [113]. The mechanism, presented in multiple studies [114] uses the phytocomponents both as reduction and capping agents. This alternative method of nanoparticles synthesis leads to materials with enhanced properties, valuable for a series of medical and industrial applications [113,115], enhancing the intrinsic properties of the nanoparticles [116], as well as a potential reduction of their toxicity [114]. The application was explored for *P. major* aqueous leaves extracts, leading to the synthesis of silver nanoparticles (AgNPs) and iron oxide nanoparticles (IONPs—spherical, 4.6–30.6 nm) and the exploration of their environmental applications, for the enhanced phytoremediation of soil and water contaminated with the insecticide fipronil [117] and for the removal of methyl orange dye, respectively [118].

## 4. Conclusions

The literature study revealed the under-utilization of the hoary plantain, a surprising aspect, considering its widespread. If the composition of *Plantago media* L. is rather well established, its applications are not nearly studied as for other *Plantago* species. By comparing the results obtained using the hoary plantain with other species belonging to the *Plantago* genus, it can be observed that the applications evaluated are only related to the biomedical field, and only trough in vitro assays. They should be further developed using in vivo assays, as should other biomedical potential applications should be explored. Also, the use of *P. media* and its natural products should be explored for further important applications, such as industrial applications (for development of food or personal care products), for pisciculture or zootechny, for phytoremediation and other environmental protection applications, or even for nanotechnological uses (a field with tremendous potential, as the phytosynthesis of different materials could find application in several areas).

## Figures and Tables

**Table 1:** General composition of *Plantago media* L., according to cited literature data.

Identified Compounds	Reference	Identified Compounds	Reference
*Polysaccharides*		*Flavonoids*	
Galactose	[30]	Luteolin	[37]
Arabinose	[30]	Apigenin	[37]
Xylose	[30]	Kaempferol	[37]
Mannose	[30]	*Phytosterols*	
Glucose	[30]	Campesterol	[32]
Rhamnose	[30]	Stigmasterol	[32]
Fucose	[30]	Sitosterol	[32]
*Iridoids*		*Fatty acids*	
Aucubin	[38]	Linolenic acid	[32]
Melittoside	[38]	Linoleic acid	[32]
Monomelittoside	[38]	Hexadecatrienoic acid	[33]
10-acetylmonomelittoside	[38]	Palmitic acid	[33]
10-acetylaucubin	[8]	Myristic acid	[33]
Catalpol	[39]	Palmitoleic acid	[33]
*Hydroxycinnamic acids*		Behenic acid	[33]
Caffeic acid	[30]	Erucic acid	[33]
Chlorogenic acid	[30]	*Carotenoids*	
Ferulic acid	[37]	β-carotene	[40]
Gallic acid	[37]	Violaxanthin	[36]
Neochlorogenic acid	[37]	Lutein	[40]
Isochlorogenic acid	[37]	Neoxanthin	[40]
*Glycosides*		Zeaxanthin	[36]
Verbascoside	[30]	*Other compounds*	
Plantamajoside	[30]	Oxalic acid	[33]
Homoplantaginin	[41]	Vitamin C	[33]
Martynoside	[42]	Ursolic acid	[43]

**Table 2:** Biomedical properties of *Plantago media*.

Plant Parts Used	Plant Treatment/Applied as	Application	Results	Reference
Aerial parts	Maceration (80% methanol, 72 h, room temperature), filtration, evaporation; extract redissolved in water (1 g/mL), nonpolar compounds removed with and concentrated—17.6% yield. Redissolved in 80% aqueous methanol for application (20% (*w*/*v*))	Antioxidant	DDPH assay: IC_50_ = 5.77 mg/L; HRS assay: IC_50_ = 271.08 mg/L; SASC assay: IC_50_ = 56.20 mg/L; NOSC assay: IC_50_ = 1.48 mg/L; FRAP assay: IC_50_ = 120.02 mg AAE/g d.w.; LP assay: IC_50_ = 31.95 mg/L;	[59]
Leaves	50% EtOH extract (100 °C, 60 min)	Antioxidant	CUPRAC assay: 0.2368 μmol AAE/g d.w.	[60]
Leaves	Methanol extract (no details provided)	Antioxidant	SOD assay: 9/17/17 activity unites/mg protein (vegetative/flowering/fruiting phase) POD assay: 1.9/2.5/0.8 activity unites/mg protein (vegetative/flowering/fruiting phase)	[61]
Leaves	Ethanol extract (no details provided)	Antioxidant	DPPH assay: 75.48% CUPRAC assay: 69.10 μM TE/g d.w. FRAP assay: 159.48 μM TE/g d.w.	[31]
Leaves	Hydrolyzed in the presence of hemicellulase enzymes (hemicellulose-H/xylanase-X, 4 h, 45 °C), filtered, coagulated (95% ethanol)	Antioxidant	DPPH assay: 84.49(H)/82.64(X)% CUPRAC assay: 64.32(H)/68.91(X) μM TE/g d.w. FRAP assay: 118.29(H)/135.69(X) μM TE/g d.w.	[62]
Leaves	Ethanol (50%) extract (solid: liquid ratio 1:20), 30 min., room temperature	Antioxidant	DPPH assay: 155 μM TE/g d.w. CH assay: 161.40 μM TE/g d.w. ORAC assay: 1274 μM TE/g d.w.	[63]
Leaves	Percolation (70% ethanol, 72 h.)	Antioxidant	DPPH assay: ~2.2 mg QE/g plant ABTS assay: ~275 μg TE/g plant	[64]
Isolated compounds	Individual compounds (verbascoside, homoplantaginin) evaluation	Antitumoral, tyrosine kinase inhibitor	Significant inhibition of isolated EGF-R tyrosine kinases, variable in vitro antiproliferative activity	[40]
Leaves	From the biomass without alcohol-soluble components: extraction with water (1:25, followed by extraction with oxalic acid/ammonium oxalate solutions (0.5%, 1:20); extract was concentrated and dialyzed; undialyzed residue precipitation by HCl (1%) in EtOH (95%) (1:5). Precipitates were centrifuged, washed (EtOH), and dried to result pectinic substances (PS) phase. Purified PS phase—raw material without alcohol-soluble components was concentrated, dialyzed, precipitated, washed, dried followed by low-molecular-weight glucans removal and precipitation with acetone	Anti-atherogenic activity	Precent binding of ALP relative to the control = 42.77/35.2% (at 20 mg/mL)	[30]
Leaves	Repeated alcohol extraction (1:5) from dried material (60% ethanol, 60 °C) followed by lyophilization (alcoholic/lyophilic extract)	Mycostatic activity	Disc-diffusion assay against *Candida albicans*, *C. utilis*, *Malas-sezia* sp., *Rhodotorula rubra*, *Aspergillus oryzae*, *A. niger*, *Microsporum canis*, *Trichophyton rubrum*, *Epidermophyton Kaufmann-Wolf*. IZ (alcoholic, mm) = 10/6/13/3/18/0.8/4.4/1.1/4.2; IZ (lyophilic, mm) = 9.2/6/2.8/2.5/17.4/0.6/4.4/1/3.2	[37]
Leaves	Methanol extraction (80%, solid: liquid ratio 1:10, 72 h., room temperature)	Anti- inflammatory activity	LPS-stimulated monocytes (U937 cell line). Evaluation of PGE_2_, TXA_2_ = 70/70% (compared with control); qPCR examination of PLA_2_, COX-1, COX-2, mPGES-1, mPGES-2, cPGES, TXAS: upregulation of COX-1, mPGES-1, TXAS; downregulated COX-2, mPEG-2, cPGES, did not influenced PLA_2_;	[43]
Leaves	Methanol extraction (80%, solid: liquid ratio 1:10, 72 h., room temperature)	Cytotoxic potential	Trypan blue exclusion test on monocytes: no impact on cell viability at up to 0.5 mg/mL	[43]

where: AAE—ascorbic acid equivalents; ABTS—2,2′-azino-bis(3-ethylbenzothiazoline-6-sulfonic acid; ALP—alkaline phosphatase; COX-1—cyclooxygenase 1; COX-2—cyclooxygenase 2; cPGES—cytosolic prostaglandin E2 synthase; CUPRAC—cupric-reducing antioxidant capacity; CH—chemiluminescnece; DPPH—2,2-diphenyl-1-picrylhydrazyl; d.w.—dry weigth; EtOH—ethanol; FRAP—ferric ion reducing antioxidant power; HRS—hydroxyl radical scavenger; IC_50—_concentration of extract leading to a 50% inhibition; IZ—inhibition zone; LP—lipid peroxidation; LPS—lipopolysaccharide; mPGES-1—microsomal prostaglandin E synthase-1; mPGES-2—microsomal prostaglandin E synthase-2; NOSC—NO scavenger capacity; ORAC—oxygen radical absorbance capacity; PGE_2_—prostaglandin E2; PLA_2_—phospholipase A2; POD—Peroxidase activity; QE—quercetin equivalents; qPCR—quantitative polymerase chain reaction; SASC—superoxide anion scavenger capacity; SOD—superoxide dismutase activity; TE—Trolox equivalents; TXA_2_—thromboxane A2; TXAS—thromboxane A synthase; U937—human myeloid leukaemia cell line.

**Table 3:** Examples of *Plantago* sp. Applications—starting point for future *P. media* studies.

Species	Product	Application	Reference
*Plantago albicans* L.	Leaves extract, (dichloromethane)	Antioxidant, anti-obesity	[85]
*Plantago coronopus* L.	Leaves and flowers, organic and water extracts	Antioxidant	[119]
*Plantago lanceolata* L.	Leaves, aqueous, ethanolic, aqueous-glycerine, and aqueous-glycol extracts	Antioxidant	[99]
*Plantago major* L.	Aerial parts, defatted aqueous methanolic extract	Antioxidant, anti-inflammatory, and hepatoprotective	[70]
*Plantago ovata* Forssk	Husk and seeds polysaccharide fraction	Antioxidant and anti-carcinogenic	[77]
*Plantago ovata* Forssk	Husk mucilage	Antioxidant and hepatoprotective (CCl_4_-induced)	[78]
*Plantago squarrosa* Murray	Whole plant, macerated in methanol (70%)	Antioxidant, antimicrobial	[67]
*Plantago major* L.	CO_2_ extract	Antimicrobial	[68]
*Plantago asiatica* L.	Whole plant aqueous extract	Anti-viral (respiratory syncytial virus)	[69]
*Plantago lanceolata* L.	Leaves, n-hexane insoluble fraction of dichloromethane extract	Anti-inflammatory	[71]
*Plantago major* L.	Aerial parts, Soxhlet extraction (benzene, chloroform, ethanol, methanol)	Anti-inflammatory	[72]
*Plantago major* L.	Leaves, aqueous and ethanol extract	Anti-inflammatory	[73]
*Plantago lanceolata* L.	Leaves, methanolic extract	Cytotoxic (tumoral cell lines)	[74]
*Plantago major* L.	Whole plant, aqueous and alcoholic extract	Antiproliferative activity (tumoral cell lines)	[75]
*Plantago major* L.	Whole plant, 80% methanol extract	Cytotoxic and genotoxic activity (*A. cepa* assay)	[76]
*Plantago ovata* Forssk	Seeds, aqueous extract	Antiulcer and hepatoprotective	[79]
*Plantago asiatica* L.	Seeds, polysaccharide fraction	Hepatoprotective	[80]
*Plantago psyllium* L.	Seeds, ethanolic extract	Hepatoprotective	[81]
*Plantago albicans* L.	Leaves, aqueous extract	Hepatoprotective	[82]
*Plantago major* L.	Whole plant, Soxhlet ethanol (70%) extraction	Renoprotective (Cisplatin induced)	[83]
*Plantago major* L.	Whole plant, Soxhlet ethanol (70%) extraction	Renoprotective (Cisplatin induced)	[84]
*Plantago major* L.	Soxhlet ethanol (70%) extraction	Renoprotective (Adriamycin induced)	[85]
*Plantago asiatica* L.	Seeds, reflux extractions	Anti-obesity	[87]
*Plantago asiatica* L.	Seeds, reflux extraction	Anti-obesity	[88]
*Plantago depressa* Willd.	Seeds, reflux extraction	Anti-obesity	[88]
*Plantago major* L.	Flavonoid fraction	Antiarrhythmic	[89]
*Plantago major* L.	Leaves extract, 70% ethanol, percolation	Anxiolytic	[90]
*Plantago asiatica* L.	Arabinoxylan (polysaccharide) isolated from the seeds	Anti-diabetic	[91]
*Plantago* sp.	Arabinoxylan (polysaccharide) extracted from seed husk	Prebiotic	[92]
*Plantago asiatica* L.	Seeds, reflux extraction	Antihypertensive	[93]
*Plantago asiatica* L.	Seeds, polysaccharide fraction	Reproductive system injury alleviation	[94]
*Plantago rugelii* Decne	Whole plant, methanol maceration (72 h.)	Anti-ulcer	[95]
*Plantago ovata* Forssk	Seeds mucilage	Lead biosorbent	[96]
*Plantago lanceolata* L.	Leaves, 40% methanol percolation, 72 h.	Pisciculture applications	[97]
*Plantago lanceolata* L.	Whole plant, acteoside and aucubin	Livestock feed	[98]
*Plantago lanceolata* L.	Leaves, aqueous, ethanolic, aqueous-glycerin, and aqueous-glycol extracts	Development of natural cosmetics	[99]
*Plantago major* L.	Seeds, gum fraction	Emulsifying and foaming properties	[100]
*Plantago major* L.	Seeds mucilage, hot water extraction	Edible coating	[101]
*Plantago major* L.	Seeds mucilage, ultrasound assisted extraction	Biodegradable films	[102]
*Plantago ovata* Forssk	Seeds mucilage, hot water extraction	Biocompatible nanofibers	[103]
*Plantago lanceolata* L.	Whole plant	Phytoremediation (Pb, As, Cd)	[104]
*Plantago major* L.	Whole plant	Phytoremediation (Pb)	[105]
*Plantago major* L.	Whole plant	Phytoremediation (Cypermethrin)	[106]
*Plantago lanceolata* L.	Whole plant	Livestock diet (improvement of mineral concentrations levels)	[107]
*Plantago lanceolata* L.	Whole plant	Livestock diet (reduction of N_2_O emissions)	[109]
*Plantago lanceolata* L.	Whole plant	Livestock diet (improvement of growth performance and carcass characteristics)	[110]
*Plantago psyllium* L.	Seeds mucilage	Dye removal	[111]
*Plantago ovata* Forssk	Polysaccharide fraction	Corrosion inhibitor	[112]
*Plantago major* L.	Leaves aqueous extract (100 °C, 60 min)	Phytosynthesis of AgNPs	[117]
*Plantago major* L.	Leaves aqueous extract (100 °C, 15 min)	Phytosynthesis of iron oxide NPs	[118]

## Data Availability

No new data were created or analyzed in this study. Data sharing is not applicable to this article.
